# Analysis of bacterial DNA in synovial tissue of Tunisian patients with reactive and undifferentiated arthritis by broad-range PCR, cloning and sequencing

**DOI:** 10.1186/ar2398

**Published:** 2008-04-14

**Authors:** Mariam Siala, Benoit Jaulhac, Radhouane Gdoura, Jean Sibilia, Hela Fourati, Mohamed Younes, Sofien Baklouti, Naceur Bargaoui, Slaheddine Sellami, Abir Znazen, Cathy Barthel, Elody Collin, Adnane Hammami, Abdelghani Sghir

**Affiliations:** 1 Laboratoire de Recherche 'Micro-organismes et Pathologie Humaine', EPS Habib Bourguiba, Rue El Ferdaous, 3029 Sfax, Tunisie; 2 Laboratoire de Physiopathologie des Interactions Hôte-bactérie, UPRES-EA 3432, Faculté de Médecine, Université Louis-Pasteur, rue Koeberlé, 67000 Strasbourg, France; 3 Service de Rhumatologie Hôpital Hedi Chaker, Avenue Majida Boulila, 3029 Sfax, Tunisie; 4 Service de Rhumatologie, EPS Fattouma Bourguiba, Rue 1er Juin, 5019 Monastir, Tunisie; 5 Service de Rhumatologie, EPS La Rabta, rue 7051 Centre Urbain Nord, 1082 Tunis, Tunisie; 6 CNRS-UMR 8030, CEA-Genoscope, rue Gaston Crémieux, 91000 Évry, France; 7 University of Evry Val d'Essonne, Boulevard François Mitterrand, 91025 Évry Cedex, 91000 Évry, France

## Abstract

**Introduction:**

Bacteria and/or their antigens have been implicated in the pathogenesis of reactive arthritis (ReA). Several studies have reported the presence of bacterial antigens and nucleic acids of bacteria other than those specified by diagnostic criteria for ReA in joint specimens from patients with ReA and various arthritides. The present study was conducted to detect any bacterial DNA and identify bacterial species that are present in the synovial tissue of Tunisian patients with reactive arthritis and undifferentiated arthritis (UA) using PCR, cloning and sequencing.

**Methods:**

We examined synovial tissue samples from 28 patients: six patients with ReA and nine with UA, and a control group consisting of seven patients with rheumatoid arthritis and six with osteoarthritis (OA). Using broad-range bacterial PCR producing a 1,400-base-pair fragment from the 16S rRNA gene, at least 24 clones were sequenced for each synovial tissue sample. To identify the corresponding bacteria, DNA sequences were compared with sequences from the EMBL (European Molecular Biology Laboratory) database.

**Results:**

Bacterial DNA was detected in 75% of the 28 synovial tissue samples. DNA from 68 various bacterial species were found in ReA and UA samples, whereas DNA from 12 bacteria were detected in control group samples. Most of the bacterial DNAs detected were from skin or intestinal bacteria. DNA from bacteria known to trigger ReA, such as *Shigella flexneri *and *Shigella sonnei*, were detected in ReA and UA samples of synovial tissue and not in control samples. DNA from various bacterial species detected in this study have not previously been found in synovial samples.

**Conclusion:**

This study is the first to use broad-range PCR targeting the full 16S rRNA gene for detection of bacterial DNA in synovial tissue. We detected DNA from a wide spectrum of bacterial species, including those known to be involved in ReA and others not previously associated with ReA or related arthritis. The pathogenic significance of some of these intrasynovial bacterial DNAs remains unclear.

## Introduction

Bacteria are considered to be important in the pathogenesis of several forms of arthritis, including reactive arthritis (ReA) [1] or various other forms of post-infectious arthritis [2]. ReA is defined as an inflammatory arthritis, occurring approximately 4 weeks after an infection, with no cultivable bacteria detectable in the joints [3,4]. Usually, the initial arthritogenic bacterial infection affects the urogenital tract (for example, *Chlamydia trachomatis*) or the digestive tract (*Yersinia*, *Salmonella *or *Shigella *spp., or *Campylobacter jejeuni*) [4]. ReA can also follow respiratory tract infections with *Chlamydophila pneumoniae *[5].

Many cases of ReA are preceded by infections that are asymptomatic [6]; such cases are clinically classified as undifferentiated arthritis (UA) [7,8]. This term describes patients who exhibit arthritis clinically similar to ReA, with high rates of monoarthritis or oligoarthrithis, and predominance of synovitis in the lower limbs. This has led several investigators to suggest a potential link between these two forms of arthritis, and that UA and ReA are overlapping entities. Several groups have detected *C. trachomatis *DNA in the synovium of patients with UA [9], suggesting that some of these patients may have a 'forme fruste' of ReA.

Arthritogenic bacterial DNA and RNA from *Chlamydia trachomatis*, *Chlamydophila pneumoniae*, and *Yersinia pseudotuberculosis *have been detected by PCR in synovial samples from patients with ReA and UA. Thus, micro-organisms, or components thereof, do reach the joint but are not always cultivable [2,9-12]. This suggests that inflammation at the joint is caused by an immune response to bacterial antigens [9,13]. Bacterial DNA has also been detected in synovial samples from patients with other forms of arthritis, such as rheumatoid arthritis (RA) or osteoarthritis (OA) [14-16]. Detection of nucleic acids from other bacteria (*Pseudomonas *sp., *Bacillus cereus*, *Mycobacterium tuberculosis*, or *Borrelia burgdorferi*) in synovial fluid or synovial tissue (ST) from patients with ReA or other forms of arthritis (UA, RA, or OA) has raised the question of whether non-*Chlamydia *or nonenteric bacteria may enter the synovium and cause or contribute toward synovitis [14,17-19]. However, the list of pathogens that trigger ReA is not definitively established.

Several studies have addressed this issue, using broad-range PCR and/or reverse transcription PCR systems to search for bacterial DNA and RNA in synovial samples from patients with various forms of arthritis, including ReA [12,14,17]. By cloning and sequencing the PCR products, they have shown that more than one micro-organism can be present in the same joint. In most studies, the PCR products were of sufficient length to determine the genus of the bacteria in the synovial samples, but were not long enough to identify the species level [12,17].

In this study we aimed to identify bacterial DNA in patients with ReA and UA using broad-range PCR, cloning and sequencing of almost the entire 16S rRNA gene. The use of this approach revealed the identity of potential bacterial causes and the presence of previously uncharacterized and uncultured bacterial pathogens in joint disease. Despite the frequent occurrence of genital and intestinal infections in Tunisia [20-24], no studies of ReA-related bacteria have yet been conducted in this country.

## Materials and methods

### Patients

Twenty-eight patients with knee effusion, who had given informed consent, were included in the study after approval from our institutional review board. All patients were attending one of three rheumatology hospital departments in Tunisia. ST samples were obtained by needle biopsy from six patients with ReA (six posturethritic) and nine with UA, and from a control group of seven patients with RA and six with OA. The patients' clinical features and demographic characteristics are summarized in Table 1.

**Table 1:** Demographic and clinical features of the study patients

Diagnosis (patients; n = 28)	Median disease duration (months [range])	Actual age or median age (years [range])	Sex or sex ratio (M/F)	Clinical details
ReA (n = 6)	2 (1–6)	35 (20–50)	5:1	
1		28	M	Sexually acquired ReA;*Ct *IgG positive serology^a^; *Ct*-positive PCR^b^
2		22	M	Sexually acquired ReA; *Ct *IgG positive^a^
3		40	F	Sexually acquired ReA; *Ct *IgG positive serology^a^
4		20	M	Sexually acquired ReA; *Ct *IgG positive serology^a^; B27+^c^
5		50	M	Sexually acquired ReA; *Ct *IgG positive serology^a^; *Ct *positive PCR^b^; B27+
6		30	M	Sexually-acquired ReA; *Ct *IgG positive serology^a^
UA (n = 9)	25 (2–60)	40 (22–59)	5:4	-
RA (n = 7)	66 (12–228)	44 (39–53)	2:5	-
OA (n = 6)	14 (12–24)	58 (44–70)	5:1	-

^a^Serology positivity was determined by microimmunofluorescence assay. ^b^*Chlamydia *PCR in genital swabs was determined by Cobas Amplicor PCR assay (Roche Diagnostics Molecular Systems, Inc, CA, USA). ^c^HLA-B27 positivity was determined using a microcytotoxicity assay. *Ct*, *Chlamydia trachomatis*; RA, rheumatoid arthritis; ReA, reactive arthritis; OA, osteoarthritis; UA, undifferentiated arthritis.

ReA was diagnosed according to European Spondyloarthropathy Study Group and Amor criteria [25,26]. All of the cases of ReA were acquired sexually, with arthritis occurring within 4 weeks of an urogenital infection (Table 1). UA was defined as a monoarthritis or oligoarthritis occurring without evidence of a predisposing infection in a patient in whom other known rheumatic diseases had been excluded.

ST samples were taken from the knee joint using the Parker-Pearson biopsy procedure [27]. Care was taken during and after obtaining patient samples to prevent cutaneous bacterial contamination. The skin surface was prepared with three successive betadine solution swabs, each for 2 minutes, and then with 70% alcohol for 2 minutes, before sampling. ST samples were immediately placed in sterile microcentrifuge tubes, which were closed and snap frozen in liquid nitrogen. Tubes were stored at -80°C until analysis.

### Automated DNA extraction

A DNA extraction procedure using the MagNA Pure system (Roche Molecular Biochemicals, Meylan, France) was used for all ST samples, using a pre-extraction treatment. Before MagNA Pure extraction, 500 μl lysis buffer (200 mmol/l NaCl, 20 mmol/l Tris HCl [pH 8], 50 mmol/l EDTA, and 1% SDS) and 25 μl proteinase K (10 mg/ml; Sigma, St Louis, MO, USA) were added to approximately 10 mg of ST. The mixture was then vigorously agitated and incubated at 65°C for 30 minutes or until complete dissociation of the ST fragments. The enzymatic reaction was stopped by incubation at 95°C for 10 minutes and samples were centrifuged at 10,000 *g *for 5 seconds. DNA was extracted on the MagNA Pure instrument using the MagNA Pure LC DNA isolation kit-Large Volume, in accordance with the manufacturer's instructions.

### Broad-range PCR amplification of 16S rRNA genes

The full-length 16S rRNA gene was amplified from extracted DNA with broad range primers (BAc08F: 5'-AGAGTTTGATCCTGGCTCAG-3'; and Uni 1390R: 5'-GACGGGCGGTGTGTA CAA-3'), targeting the region corresponding to nucleotides 8 to 27 and 1,390 to 1,407 of the *Escherichia coli *16S rRNA gene [28,29]. DNA was amplified in 50 μl reaction mixtures, each containing 1× Ex Taq Buffer (Takara *Ex taq*, Otsu, Shiga, Japan), 0.2 mmol/l of each primer, 2.5 mmol/l of each DNTP, 2 mmol/l MgCl_2 _and 1.25 units of Takara Ex Taq DNA polymerase (Takara *Ex taq*, Otsu, Shiga, Japan. T4 Gene 32 Protein (5 μg/μl; USB Corp, Cleveland, Ohio) was added to the PCR mix followed by 2.5 μl of DNA extract. PCR was performed as follows: initial denaturation at 94°C for 5 minutes, followed by 30 cycles of denaturation at 94°C for 1 minute, primer annealing at 59°C for 1 minute and extension at 72°C for 1.5 minutes. The final elongation step was extended to 15 minutes. PCR was carried out in a Gene-Amp PCR System 9700 (Applied Biosystems, Foster City, CA, USA). All extracts were tested undiluted, diluted 1:10 and 1:20, with or without T4 Gene 32 Protein, to avoid false-negative results. The T4 Gene 32 Protein was used to increase the yield of PCR products [30-32].

Pure DNA from either *E. coli *or *C. trachomatis *was used as a positive control for the broad-range PCR screening system. Amplification products were visualized on ethidium bromide-stained 1% Seakem GTG agarose gel (Tebu-bio, Le Perray en Yvelines, France).

Precautionary measures were taken to prevent DNA contamination during DNA extraction and manipulation. These included pipeting PCR components under a laminar flow of sterile air, using only sterile equipments, dedicated pre-PCR and post-PCR rooms, and dedicated sets of pipettes, disposable gloves, laboratory coats and non-reusable waste containers. Reagents and PCR primers were aliquoted to prevent frequent handlings. DNA extraction was performed in two separated biological hoods, which were cleaned before and after each sample preparation with 5% bleach solution. Gloves were changed between each tissue sample. DNA contamination was avoided using aeroguard filter tips (TipOne; Starlab, Bagneux, France) and individually self-sealing PCR tubes (Starlab, Bagneux, France), irradiated with UV light at 254 nm for 10 minutes to inactivate extraneous DNA. Negative controls (water during the amplification step and an uninfected mouse heart tissue sample during the extraction protocol) were included every five samples for each experiment to monitor potential contamination. If amplification occurred in any of the negative controls, the PCR was repeated [33]. All samples were amplified in duplicate to allow a large number of clones to be sequenced.

### Cloning, DNA sequencing and sequence analysis

The 16S rDNA amplicons were inserted into a vector using a cloning kit (pGEM-T vector; Promega, Madison, WI, USA), in accordance with the manufacturer's instructions. 16S rDNA-containing clones were grown in Nunc microtiter plates containing 150 μl of 2 × Luria-Bertani medium supplemented with 10% glycerol and ampicillin (100 μg/ml). Insert amplifications were performed using the GE Healthcare amplification kit by the RCA (rolling circle amplification) method (GE Healthcare, formerly Amersham). Amplicons were purified and then sequenced using the commercial BigDye Terminator v3.1 kit (Applied Biosystems) on a 3730XL sequencer (Applied Biosystems). The resulting 16S rDNA clones sequences were compared to sequences in the European Molecular Biology Laboratory (EMBL) databases using BLAST (basic local alignment search tool) and then checked for chimera using ribosomal database project II software [34].

### Stastistical analysis

Data were compared by Fisher's exact test using Epi Info software, version 6.04a (Centers for Disease Control and Prevention, Atlanta, GA, USA). *P *< 0.05 was considered to be statistically significant.

## Results

### PCR positivity by the broad-range PCR amplification system

Because PCR and extraction controls were negative, our results could be interpretated accurately. Amplification products of the 16S rRNA gene were generated from 21 of the 28 ST samples (75%) using broad-range PCR. Amplicons were detected in all samples from the six patients with ReA (100%) and nine with UA (100%). In the control group, bacterial 16S rDNA was amplified in ST samples from three of the seven patients with RA (43%) and from three of the six with OA (50%). Accordingly, the proportion of ST samples from ReA and UA patients yielding positive PCR results was significantly higher than that of positive ST samples from control group patients (100% [15/15] versus 46.2% [6/13]; *P *= 0.001). Additionally, ReA and UA samples exhibited a higher bacterial DNA load, as indicated by the signal intensity of the PCR products (Table 2). To enhance the spectrum of DNA from bacterial species detected, at least 24 individual clones from each sample were sequenced. Additional sequencing was performed if problems were encountered during the cloning of nonspecific or partial 16S rDNA products. In general, poorer DNA profiles of bacterial species were obtained from tissue samples that gave weak PCR signals (Table 2).

**Table 2:** Summary of PCR results and cloning details

Patient	PCR intensity score^a^	Total number of clones sequenced	Number of obtained bacterial DNA sequences
ReA			
1	++++	47	38
2	+++	96	36
3	+++	84	50
4	+++	104	48
5	+++	34	26
6	++++	24	24
UA			
7	++	105	48
8	++	114	41
9	++	72	42
10	++	75	25
11	++	96	46
12	++	74	34
13	++	118	42
14	++	60	28
15	++	101	49
RA			
16	++	48	40
17	+	96	35
18	+	48	11
OA			
19	+	48	31
20	+	72	18
21	+	48	5

^a^Semi-quantification of intensity of the 16S rDNA amplification products, visualized using ethidium bromide staining after agarose gel electrophoresis: '+' indicates barely visible band, and '++++' indicates maximal intensity. OA, osteoarthritis; RA, rheumatoid arthritis; ReA, reactive arthritis; UA, undifferentiated arthritis.

### Bacterial 16S rDNA sequences identified in synovial tissue samples

A broad range of DNAs from bacterial species was detected in each ST sample (Table 3). Only good quality sequences, with length ≥ 1,000 nucleotides, were analyzed. Most bacterial sequences had ≥ 97% sequence similarity with cultivated or uncultured bacteria. The per cent similarity to best fit sequence from the database, the accession number and the sequence length are listed in Table 4.

**Table 3:** Details of bacterial species-derived DNA sequences identified in each patient*

Patient	Total number of bacterial DNA sequences	DNA sequences identified in each patient
ReA		

1	38	9 × *Escherichia coli*, 5 × *Propionibacterium acnes*, 4 × *Stenotrophomonas maltophilia*, 3 × γ proteobacterium, 2 × *Afipia genosp*, 2 × *Escherichia *spp., 2 × swine manure bacterium, 2 × uncultured β proteobacterium, 2 × uncultured candidate division OP10 bacterium, 1 × *Alcaligenes faecalis*, 1 × α proteobacterium, 1 × *Brevundimonas diminuta*, 1 × *Pseudomonas *sp., 1 × *Ralstonia *sp., 1 × *Shigella *sp., 1 × *Sphingomonas *sp.
2	36	14 × *Escherichia coli*, 5 × *Bradyrhizobium elkanii*, 4 × swine manure bacterium, 3 × *Sphingomonas asaccharolytica*, 2 × *Pseudomonas poae*, 2 × *Ralstonia *spp., 2 × uncultured *Flavobacterium *spp., 2 × uncultured *Sphingobacterium *spp., 1 × *Flavobacterium mizutaii*, 1 × *Pseudomonas *sp.
3	50	8 × *Alcaligenes faecalis*, 7 × γ proteobacterium, 7 × *Stenotrophomonas maltophilia*, 6 × *Rhodococcus *spp., 6 × swine manure bacterium, 5 × *Shigella sonnei*, 5 × *Propionibacterium acnes*, 4 × unclassified proteobacteria, 2 × *Serratia proteamaculans*
4	48	25 × *Aquabacterium commune*, 4 × *Afipia genosp*, 4 × swine manure bacterium, 2 × *Escherichia *spp., 2 × γ proteobacterium, 2 × *Propionibacterium acnes*, 2 × *Stenotrophomonas maltophilia*, 1 × *Acinetobacter baumannii*, 1 × α proteobacterium, 1 × *Flavobacterium mizutaii*, 1 × *Ralstonia *sp., 1 × *Shigella flexneri*, 1 × *Variovorax *sp., 1 × uncultured candidate division OP10 bacterium
5	26	6 × *Aquabacterium commune*, 6 × γ proteobacterium, 3 × *Afipia genosp*, 2 × *Propionibacterium acnes*, 2 × *Ralstonia *spp., 2 × swine manure bacterium, 1 × *Shigella flexneri*, 1 × *Shigella *sp., 1 × *Staphylococcus haemolyticus*, 1 × *Stenotrophomonas maltophilia*, 1 × uncultured eubacterium
6	24	10 × *Escherichia coli*, 3 × γ proteobacterium, 2 × *Leucobacter luti*, 2 × *Staphylococcus *spp., 2 × swine manure bacterium, 2 × uncultured candidate division OP10 bacterium, 1 × *Ralstonia *sp., 1 × *Stenotrophomonas maltophilia*, 1 × uncultured *Sphingobacterium *sp.

UA		

7	48	7 × *Stenotrophomonas maltophilia*, 6 × swine manure bacterium, 4 × *Rhodococcus *spp., 4 × *Staphylococcus *spp., 4 × *Streptococcus infantis*, 3 × *Propionibacterium acnes*, 3 × *Bosea vestrisii*, 2 × *Afipia genosp*, 2 × *Blastococcus *spp., 2 × *Leptotrichia *spp., 1 × *Aeromonas *sp., 1 × *Actinomyces *sp., 1 × *Corynebacterium durum*, 1 × *Kingella oralis*, 1 × *Microbacterium oxydans*, 1 × *Neisseria flava*, 1 × *Pirellula *sp., 1 × *Shigella *sp., 1 × *Staphylococcus aureus*, 1 × *Streptococcus mitis*, 1 × *Streptococcus sanguinis*
8	41	13 × *Escherichia coli*, 6 × *Bradyrhizobium elkanii*, 5 × *Sphingomonas *spp., 4 × γ proteobacterium, 3 × *Enterobacter hormaechei*, 3 × *Stenotrophomonas maltophilia*, 2 × *Corynebacterium tuberculostearicum*, 1 × *Enterococcus cecorum*, 1 × *Flavobacterium mizutaii*, 1 × γ proteobacterium, 1 × uncultured soil bacterium, 1 × uncultured *Sphingobacterium *sp.
9	42	11 × *Escherichia coli*, 7 × uncultured *Sphingobacterium *spp., 4 × *Flavobacterium mizutaii*, 6 × uncultured *Flavobacterium *spp., 3 × γ proteobacterium, 2 × *Corynebacterium*, *tuberculostearicum*, 2 × Ralstonia spp., 2 × *Stenotrophomonas maltophilia*, 1 × *Paracoccus yeei*, 1 × *Pseudomonas poae*, 1 × *Shigella sonnei*, 1 × *Streptococcus mitis*, 1 × manganese-oxidizing bacterium
10	25	10 × *Escherichia coli*, 3 × uncultured *Flavobacterium *spp., 2 × uncultured *Sphingobacterium *spp., 2 × Bacteroidetes bacterium, 2 × *Flavobacterium mizutaii*, 1 × *Oxalobacter *sp., 1 × *Shigella flexneri*, 1 × *Shigella *sp., 1 × *Stenotrophomonas maltophilia*, 1 × uncultured α proteobacterium, 1 × uncultured candidate division OP10 bacterium
11	46	9 × *Escherichia coli*, 5 × *Acinetobacter *spp., 5 × *Stenotrophomonas maltophilia*, 5 × uncultured γ proteobacterium, 3 × uncultured *Sphingobacterium *spp., 3 × *Pseudomonas *spp., 2 × *Flavobacterium mizutaii*, 2 × *Propionibacterium acnes*, 2 × swine manure bacterium 37–8, 2 × uncultured *Flavobacterium *spp., 1 × *Aeromonas *sp., 1 × *Caulobacter *endosymbiont of *Tetranychus urticae*, 1 × *Acinetobacter schindleri*, 1 × manganese-oxidizing bacterium, 1 × γ proteobacterium, 1 × uncultured *Sphingobacterium *sp., 1 × unclassified proteobacterium, 1 × uncultured candidate division OP10 bacterium
12	34	13 × *Escherichia coli*, 4 × *Corynebacterium coyleae*, 3 × *Sphingomonas *spp., 2 × γ proteobacterium, 2 × *Ralstonia *spp., 2 × *Shigella *spp., 2 × swine manure bacterium, 2 × uncultured *Sphingobacterium *spp., 1 × *Flavobacterium mizutaii*, 1 × *Klebsiella *sp., 1 × *Propionibacterium acnes*, 1 × unclassified enterobacteria
13	42	18 × *Escherichia coli*, 4 × uncultured *Sphingobacterium *spp., 3 × *Stenotrophomonas maltophilia*, 2 × *Aeromonas *spp., 2 × *Flavobacterium mizutaii*, 2 × gamma proteobacterium, 2 × *Ralstonia *spp., 2 × uncultured candidate division OP10 bacterium, 1 × *Alcaligenes faecalis*, 1 × *Acinetobacter *sp., 1 × *Halomonas *sp., 1 × *Stenotrophomonas *sp., 1 × swine manure bacterium, 1 × *Sphingomonas *sp., 1 × uncultured *Flavobacterium *sp.
14	28	8 × *Escherichia coli*, 2 × *Bradyrhizobium japonicum*, 2 × γ proteobacterium, 2 × α proteobacterium, 2 × *Stenotrophomonas maltophilia*, 2 × *Sphingomonas *spp., 2 × *Corynebacterium durum*, 1 × *Achromobacter xylosoxidans*, 1 × Bacteroidetes bacterium, 1 × β proteobacterium, 1 × *Bradyrhizobium elkanii*, 1 × *Novosphingobium *spp., 1 × *Paracoccus *spp., 1 × unclassified Rhodocyclaceae, 1 × uncultured *Sphingobacterium *sp.
15	49	17 × *Escherichia coli*, 5 × *Shigella *spp., 4 × *Stenotrophomonas maltophilia*, 4 × unclassified Rhodocyclaceae, 3 × swine manure bacterium, 3 × uncultured candidate division OP10 bacterium, 2 × uncultured *Sphingobacterium *spp., 2 × uncultured *Sphingobacterium *spp., 2 × *Rhodococcus *spp., 1 × *Alcaligenes *sp., 1 × α proteobacterium, 1 × *Bradyrhizobium japonicum*, 1 × *Ralstonia *sp., 1 × *Flavobacterium mizutaii*, 1 × γ proteobacterium, 1 × *Pedomicrobium australicum*

RA		

16	40	15 × *Escherichia coli*, 5 × swine manure bacterium, 4 × *Ralstonia *spp., 3 × uncultured *Flavobacterium *spp., 2 × *Acinetobacter *spp., 2 × Antarctic bacterium, 2 × *Flavobacterium mizutaii*, 1 × *Actinomyces naeslundii*, 1 × *Alcaligenes faecalis*, 1 × Bacteroidetes bacterium, 1 × *Caulobacter *sp., 1 × *Corynebacterium aurimucosum*, 1 × *Shigella *sp., 1 × uncultured α proteobacterium
17	35	6 × *Escherichia coli*, 5 × *Shigella *spp., 4 × *Bradyrhizobium elkanii*, 4 × uncultured *Sphingobacterium *spp., 3 × *Ralstonia *spp., 3 × uncultured *Flavobacterium *spp., 2 × γ proteobacterium, 2 × swine manure bacterium, 1 × *Alcaligenes *sp., 1 × *Caulobacter *sp., 1 × *Pseudomonas *sp., 1 × *Rhodococcus fascians*, 1 × *Serratia proteamaculans*, 1 × *Streptococcus thermophilus*
18	11	7 × *Escherichia coli*, 2 × *Stenotrophomonas maltophilia*, 1 × γ proteobacterium, 1 × *Ralstonia *sp.,

OA		

19	31	7 × *Escherichia coli*, 7 × swine manure bacterium, 5 × *Alcaligenes faecalis*, 4 × *Pseudomonas poae*, 3 × *Bradyrhizobium elkanii*, 2 × *Stenotrophomonas maltophilia*, 1 × *Caulobacter leidyia*, 1 × *Curvibacter gracilis*, 1 × γ proteobacterium
20	18	7 × *Escherichia coli*, 7 × uncultured *Flavobacterium *spp., 2 × uncultured *Sphingobacterium *spp., 1 × uncultured delta proteobacterium, 1 × *Shigella *sp.
21	5	2 × *Alcaligenes *spp., 1 × *Achromobacter xylosoxidans*, 2 × Brucellaceae bacterium

**Table 4:** Bacterial species identified by sequencing of cloned 16S rDNA

Bacterium-derived DNA identified in ST samples	Number of patients in whom bacterial DNAs were detected	Accession number^a^	Length of the sequence^b^	% Similarity^c^
**Bacteria identified in ReA and UA patients (n = 68)**				
Bacteria previously detected in arthritis				
*Acinetobacter baumannii*	(1 ReA)	AY738400	1,384	99.86
*Acinetobacter schindleri*	(1 UA)	AJ278311	1,367	98.83
*Actinomyces *sp.	(1 UA)	AY008315	1,420	98.73
*Aeromonas *sp.	(1 UA)	U88656	1,396	99.36
*Aeromonas *sp.	(2 UA)	AF099027	1,336	98.58
*Afipia genosp 7*	(1 UA)	U87773	1,336	97.38
*Afipia genosp 9*	(1 ReA)	U87779	1,335	99.62
*Afipia genosp 9*	(1 ReA)	U87775	1,256	97.00
*Afipia genosp 9*	(1 ReA)	U87777	1,337	99.55
*Corynebacterium coyleae*	(1 UA)	X96497	1,360	99.04
*Escherichia coli*	(1 ReA)	AP009048	1,389	99.71
*Escherichia *sp.	(2 ReA)	DQ337503	1,390	99.71
*Klebsiella *sp.	(1 UA)	U32868	1,387	99.57
*Neisseria flava*	(1 UA)	AJ239301	1,338	98.43
*Paracoccus *sp.	(1 UA)	AY745834	1,308	99.92
*Propionibacterium acnes*	(1 ReA+ 2 UA)	AB108477	1,377	100.00
*Pseudomonas *sp.	(1 UA)	DQ079062	1,388	98.63
*Ralstonia *sp.	(1 ReA)	DQ227340	1,382	100.00
*Rhodococcus *sp.	(2 UA)	AF420412	1,365	99.85
*Shigella flexneri*	(2 ReA + 1 UA)	X96963	1,389	99.71
*Shigella sonnei*	(1 ReA)	X96964	1,389	99.86
*Shigella sonnei*	(1 UA)	CP000038	1,390	97.70
*Sphingomonas *sp.	(2 UA)	AJ864842	1,329	99.77
*Staphylococcus aureus*	(1 UA)	L37597	1,400	99.93
*Staphylococcus haemolyticus*	(1 ReA)	AP006716	1,400	99.93
*Staphylococcus *sp.	(1 ReA)	AJ704792	1,400	99.72
*Staphylococcus *sp.	(1 UA)	AB177642	1,399	99.71
*Stenotrophomonas *sp.	(1 UA)	AF409004	1,382	99.13
*Streptococcus infantis*	(1 UA)	AY485603	1,385	99.64
*Streptococcus mitis*	(2 UA)	AY005045	1,385	99.57
*Streptococcus sanguinis*	(1 UA)	AF003928	1,397	99.79
Bacteria not previously detected in arthritis				
*Bosea vestrisii*	(1 UA)	AF288302	1,336	99.70
*Brevundimonas diminuta*	(1 ReA)	X87274	1,308	99.62
*Corynebacterium durum*	(2 UA)	AF537593	1,364	99.05
*Corynebacterium tuberculostearicum*	(2 UA)	AJ438044	1,368	99.71
*Enterococcus cecorum*	(1 UA)	AF061009	1,398	99.43
*Enterobacter hormaechei*	(1 UA)	AY995561	1,395	99.64
*Kingella oralis*	(1 UA)	L06164	1,389	98.78
*Leptotrichia *sp.	(1 UA)	AY008309	1,359	99.85
*Microbacterium oxydans*	(1 UA)	AJ717356	1,374	98.69
*Oxalobacter *sp.	(1 UA)	AJ496038	1,387	98.05
*Paracoccus yeei*	(1 UA)	AY014169	1,309	99.77
Bacteria not previously detected in humans				
*Aquabacterium commune*	(2 ReA)	AF035054	1,367	99.85
*Blastococcus *sp.	(1 UA)	AJ316573	1,357	97.27
*Bradyrhizobium japonicum*	(2 UA)	BA000040	1,333	99.17
*Halomonas *sp.	(1 UA)	AJ302088	1,389	98.85
*Leucobacter luti*	(1 ReA)	AM072819	1,369	98.39
Novosphingobium sp.	(1 UA)	AB177883	1,335	97.00
*Pedomicrobium australicum*	(1 UA)	X97693	1,324	98.64
*Pirellula *sp.	(1 UA)	X81945	1,322	96.14*
*Sphingobacterium asaccharolytica*	(1 ReA)	Y09639	1,324	98.11
*Variovorax *sp.	(1 ReA)	AB196432	1,383	99.28
Uncultured bacteria				
α Proteobacterium	(1 ReA)	AY162046	1,308	99.62
α Proteobacterium	(1 ReA+ 21 UA)	AY162053	1,332	99.85
β Proteobacterium	(1 UA)	AF236007	1,371	99.71
*Caulobacter *endosymbiont of *Tetranychus urticae*	(1 UA)	AY753176	1,334	99.63

γ Proteobacterium	(1 ReA)	AY162032	1,395	97.42
Manganese-oxidizing bacterium	(2 UA)	U53824	1,320	99.85
Uncultured α proteobacterium	(1 UA)	AF445680	1,329	97.06
Uncultured β proteobacterium	(1 ReA)	AF445700	1,372	99.78
Uncultured candidate division OP10 bacterium	(3 ReA + 4 UA)	AF418946	1,297	90.98*
Uncultured γ proteobacterium	(1 UA)	AJ318146	1,397	97.35
Uncultured γ proteobacterium	(1 UA)	AF324537	1,387	99.71
Unclassified Enterobacteriaceae	(1 UA)	AY375058	1,391	97.56
Uncultured eubacterium	(1 ReA)	AJ292601	1,334	96.93
Uncultured soil bacterium	(1 UA)	AF423262	1,295	96.91
Unclassified proteobacteria	(1 ReA)	AY820722	1,383	96.46*
Unclassified Rhodocyclaceae	(2 UA)	AY328759	1,388	99.42
**Bacteria identified in control group (RA and OA patients; n = 12)**				
Bacteria previously detected in arthritis				
*Actinomyces naeslundii*	(1 RA)	AJ234050	1,392	97.84
Brucellaceae bacterium	(1 OA)	AY353698	1,333	99.17
*Corynebacterium aurimucosum*	(1 RA)	AY536427	1,369	99.34
*Streptococcus thermophilis*	(1 RA)	AY188354	1,397	99.36
Bacteria not previously detected in arthritis				
*Alcaligenes *sp.	(1 OA)	AF430122	1,386	98.63
*Caulobacter *sp.	(2 RA)	AJ227775	1,323	99.70
Bacteria not previously detected in humans				
*Caulobacter leidyia*	(1 OA)	AJ227812	1,324	100.00
*Curvibacter gracilis*	(1 OA)	AB109889	1,379	99.71
*Rhodococcus fascians*	(1 RA)	Y11196	1,365	99.71
Uncultured bacteria				
Antarctic bacterium	(1 RA)	AJ440974	1,321	98.86
Uncultured α proteobacterium	(1 RA)	AJ604541	1,324	98.60
Uncultured δ proteobacterium	(1 RA)	AY921777	1,402	97.22
**Common bacteria^d ^(n = 20)**				
Bacteria previously detected in arthritis				
*Achromobacter xylosoxidans*	(1 UA + 1 OA)	AF439314	1,378	99.71
*Acinetobacter *sp.	(2 ReA + 1 RA)	Z93442	1,365	99.35
*Alcaligenes faecalis*	(2 ReA+ 1 UA+ 1 RA+ 1 OA)	AY548384	1,385	99.93
*Escherichia coli*	(3 ReA+ 7 UA+ 1 RA+ 2 OA)	V00348	1,393	100.00
*Escherichia coli*	(3 ReA+ 9 UA+ 3 RA+ 2 OA)	U00096	1,390	100.00
*Flavobacterium mizutaii*	(2 ReA + 7 UA + 1 RA)	AJ438175	1,384	94.44*
*Pseudomonas *sp.	(2 ReA + 1 UA + 1 RA)	AJ237965	1,376	99.56
*Stenotrophomonas maltophilia*	(3 ReA + 4 UA + 1 OA)	AJ293470	1,395	99.93
*Shigella *sp.	(2 ReA+ 4 UA+ 2 RA+ 1 OA)	DQ337523	1,392	99.93
Bacteria not previously detected in arthritis				
*Alcaligenes *sp.	(1 UA + 1 OA)	AY672759	1,345	99.11
*Bradyrhizobium elkanii*	(1 ReA+ 2 UA+ 1 RA+ 1 OA)	AY904749	1,338	99.93
*Pseudomonas poae*	(1 ReA + 1 OA)	AJ492829	1,386	99.93
*Ralstonia *sp.	(5 ReA + 4 UA + 3 RA)	AB045276	1,388	100.00
*Serratia proteamaculans*	(1 ReA + 1 RA)	AJ233435	1,387	97.76
Bacteria not previously detected in humans				
Uncultured bacteria				
Bacteroidetes bacterium	(2 UA + 1 RA)	AY395022	1,196	97.49
γ proteobacterium	(2 ReA + 3 UA + 1 OA)	AY162042	1,399	99.88
γ proteobacterium	(4 ReA + 6 UA + 2 RA)	AY162068	1,397	99.93
Swine manure bacterium	(6 ReA+ 5 UA+ 2 RA+ 1 OA)	AY167969	1,388	100.00
Uncultured *Flavobacterium *sp.	(1 ReA+ 4 UA+ 2 RA+ 1 OA)	DQ168834	1,193	97.15
Uncultured *Sphingobacterium *sp.	(2 ReA+ 8 UA+ 1 RA+ 1 OA)	AB076874	1,390	94.31*

Number in brackets after species names indicate the number of patient set from whom bacteria were detected. ^a^Accession number of the bacterial species in the database. ^b^Length of alignment on which the 16S rDNA inserted sequence and the corresponding sequence in the database are similar. ^c^In the '% similarity' column, asterisks indicate highligh instances where the % similarity is below 97%. ^d^The 'Common bacteria' row shows the bacteria identified in ReA, UA, RA and OA patients. OA, osteoarthritis; RA, rheumatoid arthritis; ReA, reactive arthritis; ST, synovial tissue; UA, undifferentiated arthritis.

DNA from a total of 68 individual bacterial species were detected in ST samples from the patients with ReA and UA, and 12 DNAs from different bacteria were identified in the control ST samples. Additionally, DNAs from 20 bacterial species were detected in both study and control samples from patients with ReA, UA, RA, or OA. Therefore, these organisms are probably common in joint diseases (Table 4). Many sequences were from commensal bacteria, in particular those normally found in the skin or the intestinal tract (*Propionibacterium acnes*, *E. coli *and other coliform bacteria). We also detected bacterial DNAs from mucosal bacterial flora such as streptococci, *Actinomycetes *and *Neisseria*, and DNAs from opportunistic pathogens such as *Stenotrophomonas maltophilia*, *Alcaligenes faecalis*, *Achromobacter xylosoxidans *and *Acinetobacter *spp. in a number of samples. We found DNAs from organisms that are commonly identified as triggering ReA, such as *Shigella flexneri *and *Shigella sonnei *[35,36], in 33.33% of ReA and UA samples, but not in control samples. *S. sonnei *DNA was detected in samples from one ReA and one UA patient. *S. flexneri *DNA was detected in samples from two patients with ReA and one with UA. DNA from *Propionibacterium acnes *– an arthritogenic agent involved in SAPHO (synovitis, acne, pustulosis, hyperostosis, and osteitis) syndrome, which is an oligoarthritis associated with acnes and pustilosis [37,38] – was detected in ReA and UA samples. Detection of this bacterium-derived DNA was associated with *S. sonnei *(patient 3) and with *S. flexneri *(patient 5). Patient 5 exhibited pustilosis lesions associated with an urogenital infection-associated arthritis. Despite there being no history of septic arthritis in his clinical records, we detected DNAs from *Staphylococcus aureus *and streptococcal species in the ST sample from patient 7 (a patient with UA).

No genitourinary tract bacterial sequences (for example, *C. trachomatis*) were detected in our patient samples. This was unexpected, especially in ReA patients with a preceding urogenital infection. We also detected DNAs of several bacterial species that have previously been described in human infections but not in arthritis (Table 4). These include DNAs from *Bosea vestrisii*, *Brevundimonas diminuta*, *Corynebacterium tuberculostearicum*, *Corynebacterium durum*, *Microbacterium oxydans*, *Oxalobacter *spp., *Paracoccus yeei*, *Leptotrichia *spp., *Enterobacter hormachei*, *Enterobacter cecorum*, *Serratia proteamasculans *and *Ralstonia *spp. Most of these DNAs were mostly detected in one or more ReA or UA samples but not in control group samples. DNAs from *Serratia proteamasculans *and *Ralstonia *spp were also detected in the control group. Additionally, we detected DNAs from several bacterial species that have not previously been reported in human infection (Table 4). *Aquabacterium commune*, *Blastococcus *spp., *Halomonas *spp., *Leucobacter lutti*, *Novosphingobium *spp., *Pedomicrobium australicum*, *Variovorax *spp., *Sphingobacterium asaccharolytica *and manganese-oxidizing bacteria were identified from ReA and UA patient samples. We detected DNA from *Caulobacter leidyia*, *Curvibacter gracilis *and *Rhodococcus fasciens *in control group samples. We detected in ST samples some bacterial DNA sequences not previously characterized by rDNA sequencing since they exhibit less than 97% similarity to known database sequences. For example, DNA from the candidate division OP10 bacterium was detected in three ReA patients and four UA patients, but not in control group. We could find no clear association between the presence of these bacterial DNA and clinical symptoms.

## Discussion

We investigated the presence of bacterial DNA in ST samples from patients with ReA and UA, using 16S rRNA PCR, cloning and sequencing. This is, to our knowledge, the first study using the full-length 16S rRNA gene as a target for broad-spectrum PCR to detect bacterial DNA in synovial samples.

We extracted DNA from ST samples from 28 patients with arthritis. We found bacterial DNA in 21 (75%) of these patients, using stringent sterility and anti-contamination techniques. Previous studies, using PCR assays with universal 16S rDNA primers, identified lower proportions of human synovial samples containing bacterial DNA: 42% of synovial fluid and ST samples in one study [18], and 10% of ST samples in another [17]. Our high proportion of bacterial DNA in ST samples from our patients may be due to the use of the primer pair (Bac08F/Uni1390R) as well as the use of the T4 Gene 32 Protein, which may increase the yield of PCR products [30-32].

Sequence analysis of the PCR-positive samples revealed the presence of a mixture of bacterial DNA in synovial samples from patients with ReA, UA, RA or OA. These findings are similar to those reported in previous studies [12,14,17,39]. A significant disadvantage of broad-range PCR is the tendency to yield false-positive results [33,40]. In fact, we undertook stringent precautionary measures at each step (as presented in Materials and methods; see above) to prevent contamination. In addition, the MagNAPure system used is a rapid, closed, automated and standardized method for DNA extraction, eliminating many manual steps and thus minimizing the risk for cross-contamination. PCR and extraction controls consistently yielded negative results; thus, the PCR products detected in positive samples should derive only from tissue-associated bacterial rRNA genes.

Most commensal and environmental bacterial 16S rDNA sequences detected in our broad-range PCR analysis of synovial samples belong to species identified in previous studies [12,14,17,18]. Some of these were found in both the patients and control group (for instance, *Stenotrophomonas maltophilia and E. coli*), implying that their presence in the synovium is not disease specific; rather, they are likely to be opportunistic colonizers of tissue that was already diseased. *E. coli *DNA was detected in synovial samples from several patients (three with ReA, eight with UA, three with RA and two with OA). Other studies have demonstrated that commensal organisms such as *E. coli*, widely distributed in the human gut, can colonize inflamed joints [41-44]. However, a better understanding of the contribution made by intestinal microflora to human biology is needed to elucidate the potential role played by microflora in the pathogenesis of ReA [41-44].

We detected DNAs of some bacteria that have not previously been described in human synovial samples, such as *Blastococcus *spp., *Leucobacter lutti*, *Halomonas *spp., *Rhodococcus fascians *and manganese-oxidizing bacteria (Table 4). These organisms have an environmental source (soil, plant and water). Other 16S rRNA gene sequences showing less than 97% sequence similarity with bacterial sequences from the EMBL database and affiliated with noncultivated bacteria were detected. Thus, we found DNAs from uncultured candidate division OP10 bacteria in ReA and UA samples, but not in control samples. However, the synovium is probably an interfacial zone that can be colonized by bacterial DNAs originating from the environment and the endogenous microflora [37]; indeed, only a proportion of resident commensal micro-organisms in the gut have been identified [44].

Our approach of cloning and near full-length sequencing of bacterial 16S rDNA might confirm the presence of such DNAs of bacteria not considered to be human pathogens in the synovium. Their significance remains unclear, however, and further investigations will be required to determine the pathogenic relevance of these findings.

We detected DNA from *Shigella flexneri *and *Shigella sonnei *– micro-organisms that are known to trigger ReA – in ReA and/or UA samples but not in control samples. *Shigella *DNA positive patients had no clinical signs of previous intestinal infection with an enteric organism. These patients may have been asymptomatic, or the preceding gastrointestinal symptoms may have been mild and overlooked by the patients [6]. It is possible that enteric organisms may move from asymptomatic primer sites of infection to the synovium in such patients [6]. Most *Shigella *ReA cases are caused by *S. flexneri *[35], but sporadic cases associated with *S. sonnei *and *S. dysenteriae *have been described [4,35]. The most recent published case of *S. sonnei *related ReA was attributed to sexual transmission of the pathogen [36]. In our study, we detected *S. sonnei *DNA in one ReA patient presenting with an urogenital infection, which is consistent with the possibility that this species could be related to sexual transmission.

*Propionibacterium acnes *sequences were detected in synovial samples of one ReA patient with pustular lesions and two UA patients in whom we did not detect any other causative bacteria derived DNA. Thus, the presence of *P. acnes *DNA in these patients is likely to be either disease specific or due to opportunistic colonization of the inflamed joints. This bacterium is part of the normal skin flora. It was recently identified in articular samples from patients with SAPHO (synovitis, acne, pustulosis, hyperostosis, and osteitis) syndrome, which is often regarded to be a form of spondyloarthropathy, suggesting an infectious origin of this syndrome [37,38]. Our findings also suggest that this organism can access the joints.

ReA-related genitourinary bacterial species, including chlamydial sequences, were not detected in any patient samples, despite the large number of clones sequenced and analyzed for each sample. Thus, it is possible that not all bacterial DNAs present within the joint were detected. Indeed, even for arthritic patients with related urogenital infections, the detection of genital infectious agents by broad-range PCR may have been masked by the presence of other bacterial DNAs. Thus, bacteria appear to move from various anatomic sites such as gut to the joints. This is consistent with the detection of *E. coli *sequence in many of the synovial samples. In enteric ReA, active bowel inflammation affects the barrier function of the gut wall, allowing gut flora to access systemic sites [45]. Moreover, most of the patients were taking nonsteroidal drugs, which can impair gut permeability and mucosal competence.

We have shown that sequences from bacterial species that are known to be involved in the onset of arthritis represented a minority of the sequences detected in ST samples from patients with ReA. In addition, their presence was associated with DNAs from commensal and environmental bacterial flora. This raises the question about the role that this variety of intra-articular bacterial DNA plays in the pathogenesis of ReA and other forms of arthritides. However, detection of bacterial DNAs in the ST of patients with arthritis does not necessarily reflect the presence of complete bacterial genome, the presence of infectious bacteria or the potential of bacterial replication, or indicate whether detected DNA is related to the synovial pathology [2]. Within this context, the presence of multiple bacterial DNAs in patient joints does not substantiate a multibacterial infection that could ensue in these patients. Our detection of bacterial DNA in synovia of both control and study patient samples may indicate that a low level of 'background' bacterial DNA is usually present in synovial material and that such DNAs do not necessarily cause synovitis [17,46]. Such a variety of bacterial DNA could be due to the passive transfer of various bacterial products within phagocytic cells to the inflamed joint. Consistent with this, bacterial fragments have previously been detected – using immunohistochemical techniques – in macrophages from spleen of rats and humans and in synovium-derived macrophages from patients with various arthropathies [41,47,48]. Nonspecific migration of inflammatory cells containing bacterial particles into synovium could promote synovial inflammation [17]. On the other hand, bacterial DNA itself might trigger an immune response, and thus may induce synovitis. It has been shown that experimental intra-articular injection of some bacterial DNA (*Escherichia coli*, *Staphylococcus aureus*) or simply of nonmethylated CpG motifs is sufficient to trigger arthritis in mice [49-51]. The hypothesis is that bacterial DNA may be directly responsible for part of the synovial inflammation. This merits further investigations in humans, particularly to assess whether the quantity of bacterial DNA required to induce arthritis is comparable with the 'inoculum' observed *in vitro *in ReA and other arthritides.

## Conclusion

Broad-range PCR, sequencing and cloning are essential techniques for the characterization of the microbial environment in joints. This is the first study to use a broad-range 1,400 base pair 16S rDNA PCR coupled to automated DNA extraction to identify bacterial nucleic acid present in the joints of patients with ReA and other forms of arthritis. Our study provides a potential overall picture of the detailed presence of bacterial DNA in arthritic joint. Cloning procedures allowed the identification of known ReA-triggering organisms, unknown pathogens, and potentially novel bacterial species not previously associated with ReA and other forms of arthritis

## Abbreviations

EMBL = European Molecular Biology Laboratory; OA = osteoarthritis; PCR = polymerase chain reaction; RA = rheumatoid arthritis; ReA = reactive arthritis; ST = synovial tissue; UA = undifferentiated arthritis.

## Competing interests

The authors declare that they have no competing interests.

## Authors' contributions

MS performed the experimental work, analyzed the data and wrote the manuscript. RG conceived of the study, performed the design and coordination of the study, analyzed the data, and revised the manuscript. HF, MY, SB, NB and SS made pathological diagnosis, conducted sampling procedures, and performed clinical and rheumatological data analyses. AZ, CB and EC conducted assessment of *Chlamydia trachomatis *serology and DNA extraction. BJ and JS participated in the design and coordination of the study, and drafted the manuscript. AH and AS analyzed microbiological and sequencing data, and revised the manuscript. All authors read and approved the final manuscript.
